# Model-based quantification of metabolic interactions from dynamic microbial-community data

**DOI:** 10.1371/journal.pone.0173183

**Published:** 2017-03-09

**Authors:** Mark Hanemaaijer, Brett G. Olivier, Wilfred F. M. Röling, Frank J. Bruggeman, Bas Teusink

**Affiliations:** 1 Systems Bioinformatics, Amsterdam Insititute for Molecules, Medicines and Systems, VU Amsterdam, The Netherlands; 2 Molecular Cell Physiology, Amsterdam Insititute for Molecules, Medicines and Systems, VU Amsterdam, The Netherlands; Virginia Commonwealth University, UNITED STATES

## Abstract

An important challenge in microbial ecology is to infer metabolic-exchange fluxes between growing microbial species from community-level data, concerning species abundances and metabolite concentrations. Here we apply a model-based approach to integrate such experimental data and thereby infer metabolic-exchange fluxes. We designed a synthetic anaerobic co-culture of *Clostridium acetobutylicum* and *Wolinella succinogenes* that interact via interspecies hydrogen transfer and applied different environmental conditions for which we expected the metabolic-exchange rates to change. We used stoichiometric models of the metabolism of the two microorganisms that represents our current physiological understanding and found that this understanding - the model - is sufficient to infer the identity and magnitude of the metabolic-exchange fluxes and it suggested unexpected interactions. Where the model could not fit all experimental data, it indicates specific requirement for further physiological studies. We show that the nitrogen source influences the rate of interspecies hydrogen transfer in the co-culture. Additionally, the model can predict the intracellular fluxes and optimal metabolic exchange rates, which can point to engineering strategies. This study therefore offers a realistic illustration of the strengths and weaknesses of model-based integration of heterogenous data that makes inference of metabolic-exchange fluxes possible from community-level experimental data.

## Introduction

Microbial communities carry out important processes for the planet’s ecosystem, animal health and industrial purposes. Engineering such communities is not straightforward; they are generally composed of many interacting microorganisms, and they are highly dynamic. Developing methods that relate the systemic properties of communities to the underlying metabolic processes and interactions of the community members is a major challenge in microbial ecology. Those interactions drive community behaviour, including its (in-)stability upon environmental perturbations [1]. Metabolic interactions between community members are widespread and generally considered to be the dominating interactions [2–4].

Current approaches focus mostly on correlation and co-occurrence of species members for inference of community-interaction partners [5, 6]. These approaches importantly predict the community-interaction structure, even for large systems. It does not, however, inform us about interaction mechanisms and their importance for species survival and community properties. This can be achieved when we know the metabolic exchange fluxes between microorganisms; then we would capture both mechanism and importance of those fluxes, as those can be linked to intracellular metabolic activities and growth. With this additional information we could rationally design approaches that alter community behaviour upon external perturbations.

Unfortunately, quantifying metabolic exchange fluxes—metabolic interactions—directly from experimental data is rarely possible. Generally, only net fluxes are inferred from dynamic metabolite levels measured at the community level that result from contributions of many species. To determine the individual contributions of those species, we suggested that their metabolic capacities, expressed in terms of quantitative models, should be integrated with experimental data [7]. Here we illustrate that this indeed allows for the identification and quantification of metabolic interactions between microorganisms and that those interactions are dependent on environmental conditions.

Our approach relies on stoichiometric models of metabolism and the linkage of the metabolism of microbial species in the community. Stoichiometric modeling of the metabolism of single microorganisms have been developed in systems biology in the last two decades. Recently, such models are being considered for microbial communities [8–11], but the number of studies that combine such metabolic models with experimental data is still limited. The first study was performed by Stolyar *et al.* on a methanogenic co-culture [12] and several other co-culture studies followed [13, 14]. Also, purely computational studies investigated the potential interactions in a community [15], designed medium compositions that enforces metabolic interactions [16] or calculated biomass ratios and fluxes under balanced growth conditions of microbial communities [17]. The focus of those studies was mostly the prediction of the community phenotype. However, another application of those models is to use them to infer metabolic exchange fluxes between microorganisms from experimental data [7].

Here we demonstrate that we are able to quantify community interactions by combining experimental data, consisting of biomass abundances and metabolite concentrations, with stoichiometric metabolism models. For this purpose, we designed a synthetic co-culture that serves as a model for anaerobic interspecies hydrogen transfer, which is one of the driving forces in the anaerobic digestion process, and allows for tunable metabolic interactions by changing the environmental conditions. Quantification of the metabolic interactions contributes to identifying how this important process can be improved. Although synthetic communities are not as complex as natural ecosystems, they have shown to be useful for microbial ecologists to examine ecological theories, such as the influence of community evenness on the functionality of a community [18]. Additionally, synthetic communities are used to improve industrial applications, such as bioremediation [19] or bioethanol production [20]. The co-culture we designed consists of the anaerobic bacteria *Clostridium acetobutylicum* and *Wolinella succinogenes*. *C. acetobutylicum* produces H_2_ that subsequently is consumed by *W. succinogenes* to reduce nitrate (NO_3_^-^) into nitrite (NO_2_^-^) or ammonium (NH_4_^+^). NO_2_^-^ or NH_4_^+^ can act as a nitrogen-source for *C. acetobutylicum*. We varied the nitrogen source to vary the metabolic interactions in the community, which we subsequently inferred, using a model and experimental data. This study illustrates how the integration of a model, representing our knowledge about the metabolism of the two species, with experimental data leads to the quantification of metabolic exchange fluxes.

## Materials and methods

### Strains and cultivation conditions

Co-culture experiments of *Clostridium acetobutylicum* DSM-792 with *Wolinella succinogenes* DSM-1740 were grown in Widdel-based medium containing the following components (per liter): 1 g NaCl, 0.4 g MgCl⋅6H_2_O, 0.1 g CaCl⋅2H_2_O, 0.5 g KCl, 0.59 g glucose⋅H_2_O, 0.15 g cysteine, supplemented with 1 ml of 100x RPMI-1640 vitamin solution (Sigma-Aldrich) and 1 ml of trace elements solution, containing (per liter): 0.5 g EDTA, 3 g MgSO_4_⋅7H_2_O, 0.5 g MnSO_4_⋅H_2_O, 1 g NaCl, 0.1 g FeSO_4_⋅7H_2_O, 0.1 g Co(NO_3_)_2_⋅6H_2_O, 0.1 g CaCl_2_, 0.1 g ZnSO_4_⋅7H_2_O, 10 mg CuSO_4_⋅5H_2_O, 10 mg AIK(SO_4_)_2_, 10 mg H_3_BO_2_, 10 mg Na_2_MoO_4_⋅2H_2_O, 1 mg Na_2_SeO_3_, 10 mg Na_2_WO_4_⋅2H_2_O, 20 mg NiCl_2_⋅6H_2_O. A 20 mM Na-phosphate buffer pH 7.0 was used to maintain a constant pH during the growth experiment. The final pH of the medium was 7.0. The medium was kept anaerobic by flushing with a gas mixture of 10% CO_2_ and 90% N_2_. Resazurine (0.01 mg/l) was used as an indicator for anaerobic conditions. The growth experiment was performed at 37°C in 100 ml septum vials with rubber stoppers containing 50 ml of growth medium. ‘NH_4_^+^’ condition contained 0.25 g/L NH_4_Cl, ‘NO_3_^-^’ condition contained 0.85 g/l NaNO_3_^-^, ‘NH_4_^+^ + NO_3_^-^’ condition contained 0.25 g/l NH_4_Cl and 0.85 g/l NaNO_3_^-^ and ‘N_2_’ condition contained no N-source, except N_2_ in the headspace. Cells were taken from actively growing stock, cultivated in monoculture first in abovementioned medium, but with yeast extract supplemented. The monocultures were added 1:1 (OD/OD) in the media, pre-grown for 70 generations via serial transfer of the co-culture in NH_4_^+^ + NO_3_^-^ containing medium to create a stable co-culture. We are aware that mutations could arise during this period which potentially influence the community phenotype, but that is beyond the scope of this study. The co-culture was 1:100 propagated to fresh medium once it was fully grown. The experiment started when the co-culture reached exponential growth and was 1:100 propagated to fresh medium containing different nitrogen sources in triplicate.

### Metabolite analysis

Supernatant from the batch cultivation was taken from the septum vials by taking 1 ml of growth medium and filtered through a 0.2 μm polyethersulfone (PES) filter and stored at -20°C until further processing. Samples were analyzed for fermentation products (i.e. formate, succinate, acetate, propionate, lactate, and butyrate) by high performance liquid chromatography (HPLC) with a Phenomenex Rezex ROA 300x7.8mm column at 55°C and occupied with RID 10A and SPD 20A detectors having a flow rate of 0.5ml/min and eluent of 5mM H_2_SO_4_. NO_3_^-^ was measured according Yang et al. [21] and NO_2_^-^ was measured spectrophotometrically at 520nm by diazotization of sulphanilamide by nitrite. CO_2_ and H_2_ were measured by taking 100 μl gas sample from the head space with a syringe (Terumo 1 mL syringe) and the gas was immediately analyzed on a Shimadzu GC2010 system equipped with a Carboxen 1010 column (30m⋅0.58mm, 105°C) and a barrier discharge ionization detector (BID). Helium was used as a carrier gas at 28.2 mL/min at a pressure of 37.2 kPa.

### Quantification of cells

DNA was extracted with the PowerSoil DNA isolation kit (MO BIO Laboratories, Solana Beach, CA, USA), according the manufacturer protocol, from 2 ml of culture. Species abundance was measured by quantitative PCR using primers for *C. acetobutylicum* [22] and *W. succinogenes* (forward primer (5’→3’): CCACACGACACTACCCTCAC and reverse primer (5’→3’): CGGTGCTTACGATTCCCAGT) on a 7300 Real Time PCR System (AB Applied Biosystems, CA, USA). Both primer sets are selective for the target species and the quantification of genes is corrected with the number of genes on the genome. The following PCR program was used for *C. acetobutylicum* quantification: a start-up step of 50°C for 2 minutes, followed by an initial denaturation step at 94°C for 10 minutes, 40 cycles of 94°C for 30 seconds, 54°C for 30 seconds and 72°C for 40 seconds, plus a final elongation step at 72°C for 5 minutes. For quantification of *W. succinogenes* we used: a start-up step of 50°C for 2 minutes, followed by an initial denaturation step at 94°C for 10 minutes, 45 cycles of 94°C for 10 seconds, and 60°C for 30 seconds.

### Models and software

All simulations were done using CBMPy 0.7.0 (http://cbmpy.sourceforge.net/), a Python-based software package. The genome-scale metabolic model of *C. acetobutylicum* [13], a coarse-grained metabolic model and a genome-scale metabolic model of *W. succinogenes* (see supplements), using the SBML level 3 standards [23], were used to perform the simulations (All models and scripts are available on https://sourceforge.net/projects/cbmpy/files/publications/data/2017_Hanemaaijer/). The method to reconstruct the genome-scale metabolic model of *W. succinogenes* is described in the supplemental material. Dynamic flux balance analysis simulations were based on the method by Zhuang *et al.* [24], but implemented in Python using the LSODA integrator provided by the SciPy scientific library [25]. The CPLEX-solver (academic license) from IBM^®^ was used for optimization of the metabolic networks. The method for model reconstruction and co-culture growth simulations are described in the supplements. The Flux Variability Analysis was performed according Mahadevan *et al.* [26].

## Results

### Design of co-culture and media composition that gives rise to different metabolic interactions

**Fig 1:**
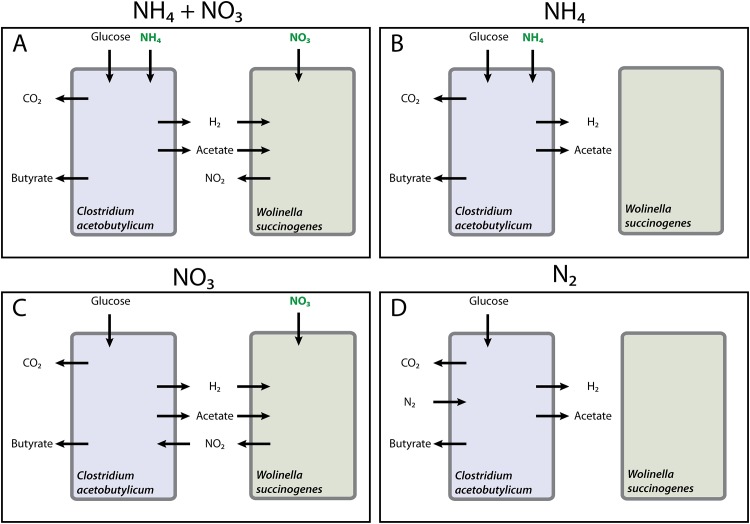
Source of nitrogen is expected to influence the metabolic interactions between *C. acetobutylicum* and *W. succinogenes*. **A** shows the expected interactions between *C. acetobutylicum* and *W. succinogenes* in the presence of NH_4_^+^ and NO_3_^-^. **B** shows the interactions in the presence of only NH_4_^+^, **C** in the presence of only NO_3_^-^ and **D** shows the expected interactions in the presence of only N_2_. Although we expect certain interactions in the co-culture during the various environmental conditions, we do not know at what rate the metabolites are exchanged.

We designed a synthetic co-culture of *C. acetobutylicum* and *W. succinogenes*, serving as a model system for anaerobic interspecies hydrogen transfer. These species are chosen, because they allow for tunable metabolic interactions by changes made to the medium composition, in particular the nitrogen source (Fig 1).

We choose *C. acetobutylicum* because it produces H_2_ from glucose, which can be consumed by *W. succinogenes* to generate energy for the reduction of NO_3_^-^ to NO_2_^-^ or, further, to NH_4_^+^ when the NO_3_^-^ concentration is low. We choose a high NO_3_^-^ concentration in our experimental set-up to prevent this. As an alternative to H_2_ *W. succinogenes* can use formate. Since *C. acetobutylicum* cannot use NO_3_^-^ as a nitrogen-source it has to fix N_2_ gas when NO_3_^-^ is the sole nitrogen source in pure culture [27]. However, in co-culture *C. acetobutylicum* can use NO_2_^-^ produced by *W. succinogenes*. Alternatively, when NH_4_^+^ is present, both organisms can use it as nitrogen source, but *W. succinogenes* would still not grow because it lacks an energy source. Varying the nitrogen source also has additional effects. When NH_4_^+^ and NO_3_^-^ is present, *C. acetobutylicum* is independent of *W. succinogenes* and supplies H_2_ to *W. succinogenes*. *C. acetobutylicum* becomes dependent on *W. succinogenes* when only NO_3_^-^ is present. Then *W. succinogenes* cross feeds NO_2_^-^ to *C. acetobutylicum*, which, in turn, supplies H_2_ that facilitates NO_3_^-^ to NO_2_^-^ reduction by *W. succinogenes*. Thus we can design co-cultures with uni- and bidirectional metabolic interactions. We studied four different conditions; the associated, expected metabolic interactions are shown in Fig 1.

### Experimental data of four growth conditions

The co-culture was grown in four different environmental conditions, in which we varied the nitrogen-source to study its influence on the state of the community, outlined in Fig 1. The media either contained NH_4_^+^ and NO_3_^-^, only NH_4_^+^, only NO_3_^-^ or neither. N_2_ was the only nitrogen-source in the absence of NH_4_^+^ and NO_3_^-^, with the expectation that only *C. acetobutylicum* can grow. Across conditions, the biomass abundances and metabolite concentrations were measured in time to study the impact of the nitrogen-source on the co-culture dynamics.

**Fig 2:**
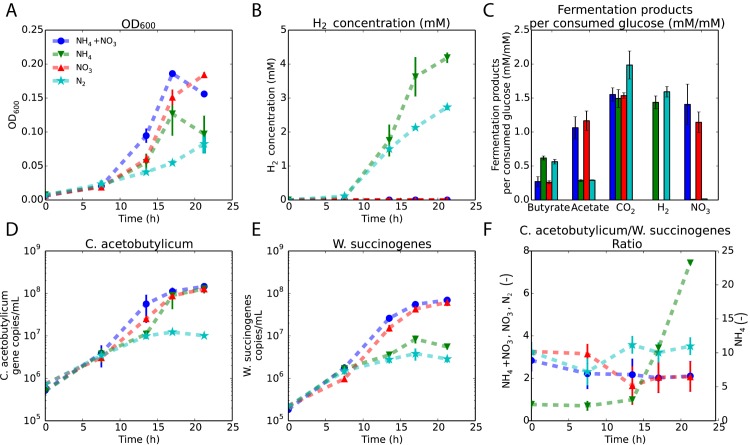
Nitrogen-source alters the phenotypic behaviour of the co-culture in batch experiments. **A** shows optical density measurements at 600 nm. **B** is the measured H_2_ concentrations. **C** are fermentation products per consumed glucose at the the end of the cultivation period and is the sum of the production and consumption of the species in the co-culture. The NO_3_^-^ is the amount of NO_3_^-^ reduced. **D** and **E** are the quantified gene copies concentration per mL of *C. acetobutylicum* and *W. succinogenes*. **F** shows the ratios of *C. acetobutylicum* and *W. succinogenes* based on gene-copy number. This is to show that both strains show balanced growth during batch cultivation. The copy numbers are corrected for the amount of genes on the genome.

The different conditions influenced the growth and metabolic profiles of the co-culture (Fig 2). The co-culture grew slower in the N_2_ condition and led to a 2-fold lower optical density than the NO_3_^-^ condition (Fig 2A). In the N_2_ condition, *C. acetobutylicum* fixes N_2_ gas to acquire nitrogen, which is an energetically unfavourable process [28, 29]. The N_2_ condition was also the only condition where glucose was not completely consumed at the end of the experiment. A drop in the optical density was observed in two conditions at the end of the experiment, because of induction of sporulation by *C. acetobutylicum*. We only detected H_2_ when NO_3_^-^ was absent (Fig 2B), suggesting that *W. succinogenes* consumed all the H_2_ produced by *C. acetobutylicum* in the presence of NO_3_^-^ (since no formate was detected). The final amounts of butyrate, acetate, CO_2_ (produced by *C. acetobutylicum*) and reduced NO_3_^-^ (by *W. succinogenes*) varied between the different conditions (Fig 2C). In the absence of NO_3_^-^, more butyrate was formed out of glucose, at the expense of acetate. This indicates a bidirectional interaction: in the presence of NO_3_^-^, when *W. succinogenes* grows, the H_2_ concentration is kept low by *W. succinogenes*, such that *C. acetobutylicum* has a different fermentation profile due to alleviation of H_2_-inhibition, which is consistent with literature [30]. This is therefore proof of a bidirectional interaction, because *C. acetobutylicum* behaves as in pure culture in the absence of NO_3_^-^, with high H_2_ and butyrate concentrations. Note that H_2_ accumulates in the absence of NO_3_^-^, because the growth rate of *W. succinogenes* decreases (Fig 2E). *C. acetobutylicum* produced 30% more CO_2_ from glucose in the ‘N_2_’ condition relative to the other conditions.

Fig 2F indicates time periods during which a constant biomass ratio of the two species were observed. Only in the presence of NH_4_^+^, *W. succinogenes* was outgrown by *C. acetobutylicum* after 13.5 hours, because no NO_3_^-^ is available for *W. succinogens* to convert H_2_. In the other cases we found constant biomass ratios, always after 13.5 hours of cultivation, indicating that the two microorganisms grew at the same specific rate. Equal specific growth rates indicate that the growth of the two organisms was coupled. Such growth coupling can either result from bidirectional exchange of growth-supporting metabolites, or a unidirectional exchange from the slower-growing to the faster-growing microorganism. A bidirectional exchange was expected in the presence of only NO_3_^-^, whereas in the presence of NH_4_^+^ and NO_3_^-^ a unidirectional exchange was expected. Coupling occurs because the microorganism that is capable of a higher growth rate becomes limited by the other organism, which cannot supply its resources fast enough.

Which metabolites are exchanged and at what rate across those conditions cannot be deduced directly from the experimental data. For instance, we cannot conclude whether *C. acetobutylicum* only consumed NH_4_^+^, or that NO_2_^-^ was also consumed in the presence of NH_4_^+^ and NO_3_^-^. Also the H_2_ production rate of *C. acetobutylicum* remains unknown, as we could not detect H_2_ in two of the four conditions. Even though we can conclude that the species do exchange metabolites with each other, as they grew equally fast, we are not sure which metabolites are being exchanged let alone at what rate. To address those questions we analysed our experimental data with metabolic models of the two species.

### Stoichiometric models of co-culture metabolism and growth

We used stoichiometric metabolic models of *C. acetobutylicum* and *W. succinogenes* to fit the experimental data for the inference of metabolic interactions between the species during different environmental conditions. The metabolic models only describe the stoichiometry of metabolic reactions, based on genomic and biochemical information, and do therefore not contain any kinetic parameters of all the individual enzymes. In addition, we took the growth rates and the biomass abundances of the microorganisms into account to capture the metabolism and the dynamics of the co-culture, using an existing method called dynamic flux balance analysis (dFBA) [31], which extends the stoichiometric model by adding kinetic information of growth-limiting substrate importers in the form of an irreversible Michaelis-Menten equation ($v_{upt}=V_{max}\frac{\left[S\right]}{K_{m}+\left[S\right]}$), where *v*_*upt*_ represents the uptake reaction, *V*_*max*_ the maximum rate of the uptake reaction, [S] the substrate concentration and *K*_*m*_ the substrate affinity. At each time step in dFBA the biomass production rate for each organism is independently optimised based on the specific substrate uptake rate. Implementing such dynamic uptake reaction enables the model to respond to changes in external concentrations.

For the simulations we use a genome-scale metabolic model for the metabolism of *C. acetobutylicum* and for *W. succinogenes* a coarse-grained model, which lumps metabolic segments into single reactions. We used a coarse-grained model as we expected that we could model our results without the need for full genome-scale reconstruction. We did do the reconstruction to verify that this was indeed the case (see supplemental material). Having a coarse-grained model facilitates computation and interpretation, especially if these methods are applied to larger and more complex microbial ecosystems.

Conversely, the detailed model of *C. acetobutylicum* has the advantage that it captures changes in its metabolism, as we observed that final acetate and butyrate concentrations, produced by *C. acetobutylicum* changes when the growth medium was varied. Another advantage is that it describes the metabolic flexibility associated with H_2_ production by *C. acetobutylicum* and leads to oxidization of ferredoxin. Ferredoxin is a conserved moiety whose total concentration does not change (only its redox state), and when it is oxidized by another reaction, it will result in decreased H_2_ production. Since *C. acetobutylicum* contains several reactions that can oxidize or reduce ferredoxin, the H_2_ production flux is flexible, influencing the community interactions that we aim to quantify. For instance, NO_2_^-^ uptake decreases the amount of H_2_ production, because both reactions require ferredoxin, which results in potential interesting dynamics between the two species, because *W. succinogenes* provides NO_2_^-^, but requires H_2_ which is produced by *C. acetobutylicum*. Several genome-scale metabolic models of *C. acetobutylicum* are available [32, 33]. We decided to use the model created by Salimi and co-workers [13], because this model was already successfully used for dFBA simulations.

We constructed a coarse-grained model of the core metabolism of *W. succinogenes*, containing eight reactions (S1 Table) that was detailed enough to fit the exchange fluxes to experimental data; the data indicated that *C. acetobutylicum* shows most metabolic flexibility. The ATP yield of NO_3_^-^ reduction, when H_2_ is oxidized, was taken from literature [34]. We fitted the elemental composition of *W. succinogenes* biomass. It can use acetate as a carbon source [35], but with an unknown biomass yield. Similarly the NH_4_^+^ and ATP requirements for the production of one unit of biomass are unclear. We estimated all these values by varying them and used those values that resulted in the best fit with the experimental data. An advantage of working with coarse-grained models is that the model complexity is greatly reduced and the degrees of freedom are limited, whereas in genome-scale metabolic models these can increase rapidly [36–38]. However, we also constructed a draft genome-scale metabolic model of *W. succinogenes* to compare the results from the simulations with this model with the coarse-grained model (S1 File).

The two metabolic models were coupled through exchange fluxes for H_2_, NO_2_^-^ and acetate. The co-culture model was manually fitted to the experimental data, using dFBA, to infer the metabolic exchange fluxes in the microbial community. Manually fitting was done by changing the kinetic parameters (S2 Table), biomass composition of *W. succinogenes* (S1 Table) and ratio of acetate/butyrate production and NH_4_^+^/NO_2_^-^ consumption until the simulation agreed most with the experimental data. ame

### The metabolic models can simulate the experimental data

Once the simulations agree with the experimental data, the corresponding metabolic fluxes can be extracted from the simulations, including the exchange fluxes between the two species. This allows us to infer the metabolic interactions between species in a microbial community.

To fit the model with the experiments, we varied the parameters of the models (S2 Table) to find an optimal fit. These parameters influence the rate of consumption and production of the metabolites in the co-cultures and can be different between the various environmental conditions. For instance, the V_*max*_ of glucose uptake in *C. acetobutylicum* changes between the different conditions (S2 Table). Although the parameters can change between the conditions, we did not change the metabolic models itself. The structure of the metabolic models, therefore, remain identical between the different environmental conditions.

For *C. acetobutylicum*, we had to constrain the ratio of butyrate and acetate production fluxes and the ratio of the NH_4_^+^ and NO_2_^-^ consumption to fit the experimental data (S2 Table). Removing those ratios resulted in incorrect simulation of the metabolic profiles, which is the result of apparent suboptimal behaviour of *C. acetobutylicum*. One reason for this could be that H_2_ inhibits certain reactions and results in suboptimal behavior. Several studies showed that H_2_ affects the metabolism of *Clostridia* species [30, 39]. An other reason could be that there is a yield/rate trade-off and a high growth rate would result in a suboptimal biomass yield as observed for other organisms. Flux balance analysis will always find the flux distribution that maximizes this yield, not the rate. We also had to fit V_*max*_ and K_*m*_ of glucose uptake by *C. acetobutylicum* and the V_*max*_ and K_*m*_ of H_2_ uptake by *W. succinogenes* (S2 Table).

**Fig 3:**
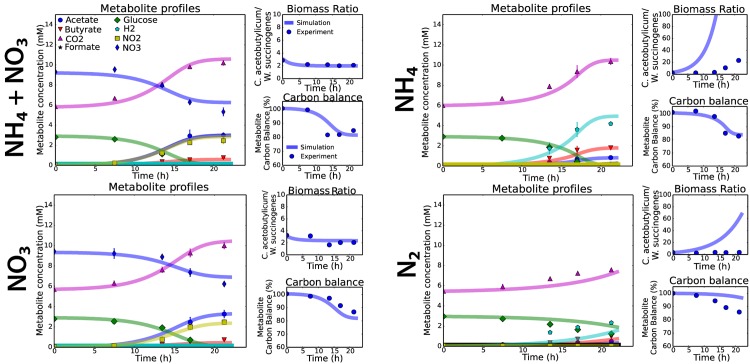
The dFBA simulations agree mostly with the experimental data for the four different cultivation conditions and were used to infer the metabolic fluxes. The metabolite profile plots contain error bars, but the other subplots not. The biomass ratios are based on the gene-copy data and the carbon balance consisted of the measured metabolites that contained carbon. The remaining missing carbon is assumed to be incorporated into biomass.

**Fig 4:**
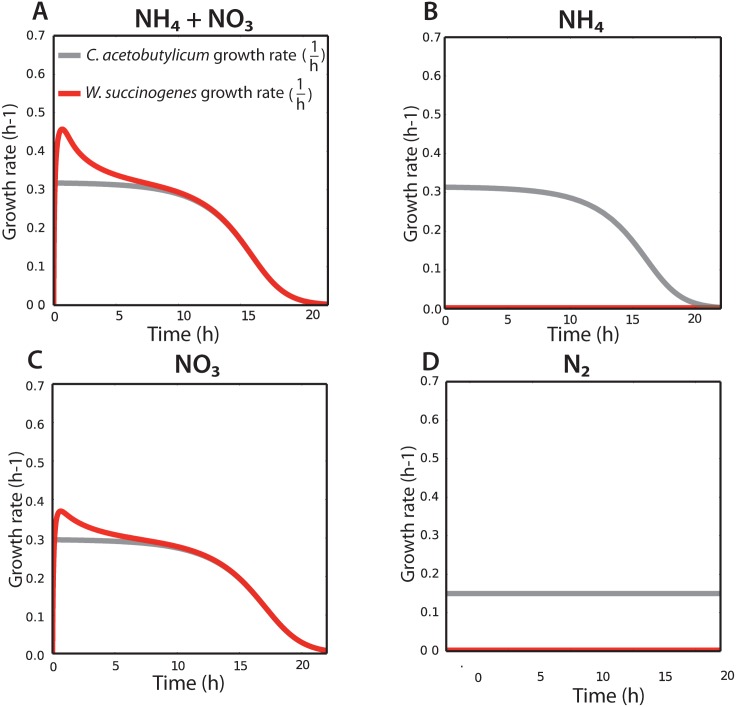
Growth of *W. succinogenes* was limited by the growth-rate of *C. acetobutylicum*, based on the inferred growth-rates. Balanced growth was observed after 10 hours of cultivation in the NH_4_^+^ + NO_3_^-^ (**A**) and NO_3_^-^ conditions (**C**). No growth of *W. succinogenes* was simulated in the NH_4_^+^ (B) and N_2_ conditions (**D**).

The simulation with the metabolic models are consistent with the experimental data, except that *W. succinogenes* grew in the absence of NO_3_^-^, which was not predicted with the model (Fig 3). This is most likely caused by carry-over of NO_3_^-^ from the pre-culture and by cell lysis. However, these phenomena were not taken into account in the simulations of the metabolic model and therefore, no growth of *W. succinogenes* was predicted with the metabolic models (Fig 4B and 4D). Simulations where the genome-scale metabolic model of *W. succinogenes* was used were similar relative to the simulations with the coarse-grained model of *W. succinogenes* (S1 File).

### Influence of environmental conditions on the metabolic exchange fluxes between *C. acetobutylicum* and *W. succinogenes*

Simulations with the fitted model indicates that the growth rates of *C. acetobutylicum* and *W. succinogenes* are dynamic during the experiment (Fig 4). The biomass yields on substrate, however, remain constant during the whole experiment (S2 Fig). The dynamic behaviour of the community resulted therefore solely from the interplay between the dynamic concentrations of the environmental nutrients and products and the biomass abundances.

*W. succinogenes* grew faster than *C. acetobutylicum* in the first 10 hours in the presence of NH_4_^+^ and NO_3_^-^. This is possible, because *C. acetobutylicum* is more abundant than *W. succinogenes* and can provide excess H_2_ despite the slower growth of *C. acetobutylicum*. After 10 hours, both species grew at the same specific rate. This is reflected in the experimental data of the biomass ratios of *C. acetobutylicum*/*W. succinogenes*, which decreased in the first hours of the experiment suggesting that *W. succinogenes* grows faster than *C. acetobutylicum*. This indicates that *C. acetobutylicum* determined the growth-rate of the community, because H_2_ consumption of *W. succinogenes* was limited by the H_2_ production of *C. acetobutylicum* after ten hours of cultivation.

**Fig 5:**
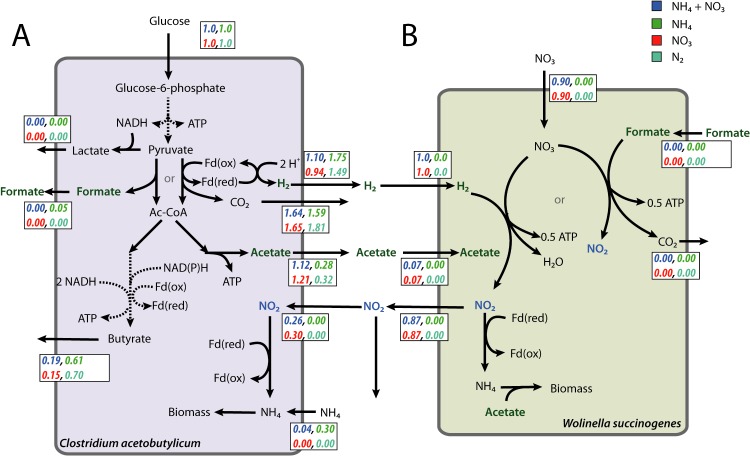
Nitrogen-source also had an impact on the H_2_ and the NO_2_^-^ exchange rates. In **A** the calculated flux-values for *C. acetobutylicum* are normalized for the glucose uptake. **B** shows the calculated flux-values for *W. succinogenes* and are normalized for the H_2_ uptake. Note that H_2_ production by *C. acetobutylicum* is not equal to the specific H_2_ uptake rate of *W. succinogenes* as the biomass abundances should be taken into account, which were not equal during the experiment.

Different nitrogen sources lead to changed metabolic interactions between *C. acetobutylicum* and *W. succinogenes* (Fig 5). The H_2_ production rate of *C. acetobutylicum*, during cultivation in the presence of NO_3_^-^, differed between the conditions: 1.10 and 0.94 mol/mol H_2_ per consumed glucose, for the ‘NH_4_^+^ + NO_3_^-^’ and ‘NO_3_^-^’ condition, respectively. H_2_ production was reduced by 17% in the ‘NO_3_^-^’ condition relative to the ‘NH_4_^+^ + NO_3_^-^’ condition. H_2_ is associated with oxidization of ferredoxin and this ferredoxin is also oxidized when NO_2_^-^ is reduced to NH_4_^+^. Since ferredoxin is a conserved moiety, an increase in the NO_2_^-^ reduction flux causes a lower H_2_ production flux, which occurred in the ‘NO_3_^-^’ condition. Therefore, there is an interesting dynamic between *C. acetobutylicum* and *W. succinogenes* when NO_3_^-^ is in the medium, because *C. acetobutylicum* can use its ferredoxin for the production of H_2_, but also for the consumption of NO_2_^-^, which is produced by *W. succinogenes*. We show that the nitrogen source can have implications for interspecies hydrogen transfer in a community. An oxidized nitrogen source, in this case NO_2_^-^, would decrease H_2_ transfer and therefore NH_4_^+^ is the preferred substrate for maximization of interspecies hydrogen transfer. Contrary to our expectations, a combination of NH_4_^+^ and NO_2_^-^ is predicted to be utilized by *C. acetobutylicum* in the NH_4_^+^ + NO_3_^-^ condition. One explanation for *C. acetobutylicum*’s utilization of NO_2_^-^ is to detoxify the medium. High NO_2_^-^ concentrations inhibit growth and utilizing NO_2_^-^ as a nitrogen source could be a detoxification strategy [40].

In contrast to the inferred fluxes of *C. acetobutylicum*, the inferred fluxes of *W. succinogenes* were equal between the ‘NH_4_^+^ + NO_3_^-^’ and ‘NO_3_^-^’ conditions (Fig 5B), suggesting that *W. succinogenes* was insensitive to the change in environmental conditions. This is primarily due to the limited flexibility of the model of *W. succinogenes*; however, the model fits with the experimental data, which suggests that the inferred fluxes are realistic. The simulations with the draft genome-scale metabolic model of *W. succinogenes* showed similar results relative to the coarse-grained model, except that the amount of consumed NO_3_^-^ and produced NO_2_^-^ per H_2_ was different (S3 Fig). This is primarily caused by the nitrogen and energy requirements for the production of biomass and is different for both models. We expect that a more accurate biomass function for the genome-scale metabolic model of *W. succinogenes* would improve the results.

### Intracellular fluxes of *C. acetobutylicum* can be inferred from community-level experimental data

In addition to calculation of the uptake and production fluxes, the genome-scale metabolic model of *C. acetobutylicum* informs us about intracellular flux values across the different conditions. This gives additional information about the active metabolic pathways. Due to the large number of reactions in the genome-scale metabolic model, the predicted values of the intracellular fluxes in the metabolic network are likely underdetermined, because we have too few experimental data to constrain those fits sufficiently.

We therefore performed a Flux Variability Analysis (FVA) [26], to check whether alternative intracellular flux values of the *C. acetobutylicum* metabolic network could fit the experimental data equally well. This analysis indicates a small flexibility, only 41 reactions of the 744 reactions had variable values (5.5%) (https://sourceforge.net/projects/cbmpy/files/publications/data/2017_Hanemaaijer/). Those 41 reactions are involved in either malate cycling, proline and nucleotide biosynthesis, and carry very small fluxes relative to the fluxes in *C. acetobutylicum*’s core-metabolism. Two reactions that did carry a high flux are involved in ferredoxin cycling, but had no impact on the H_2_ production flux. There was also no variability identified in other uptake or production fluxes. These results suggest that in our system, the calculated flux distribution within *C. acetobutylicum* can be robustly predicted from community-level experimental data alone.

### Modelling predicts that co-culture behaviour can be enhanced

Besides providing a method for inferring experimental fluxes from experimental data, the metabolic model can also be used to predict the optimal behaviour of the community when not constrained with experimental data. Optimisation informs us about optimal metabolic coupling between the microorganisms when growth of both species are optimised.

**Fig 6:**
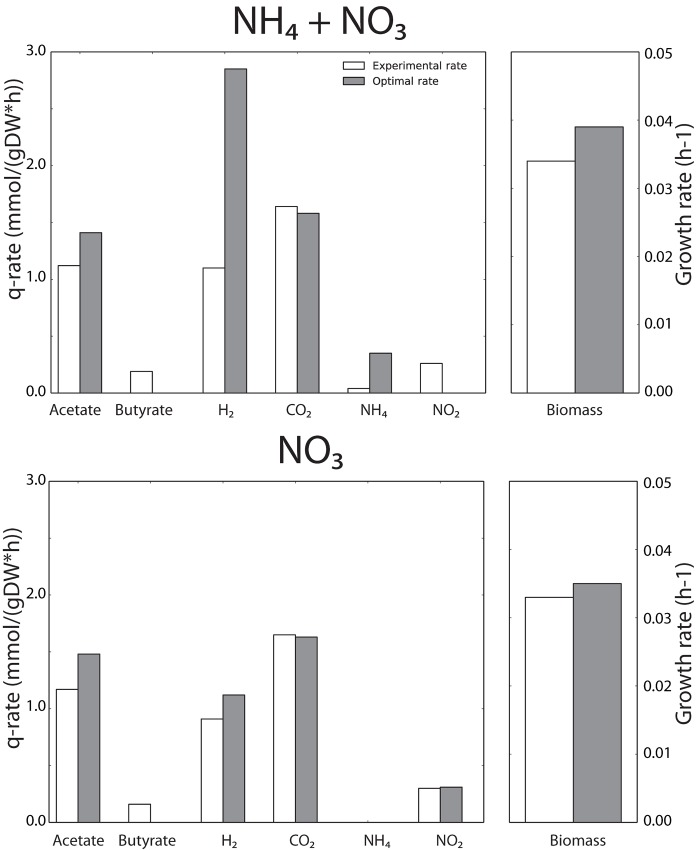
The ‘NO_3_^-^’ condition behaved more optimal than the ‘NH_4_^+^ + NO_3_^-^’ condition. The calculated experimental specific uptake and consumption rates (mmol/(gDW⋅h)) for *C. acetobutylicum* (white bars) and the *in-silico* optimal rates (gray bars) were compared. All specific uptake and production fluxes were normalized to the specific glucose uptake rate.

Here, we consider the co-culture as optimal when the growth rates of both organisms are maximal. In dFBA this means that we optimise the flux towards biomass of both organisms on the supplied nutrients to the community. We determined the metabolic fluxes (internal and exchange), growth rate and biomass abundances corresponding to the optimal state in the ‘NH_4_^+^ + NO_3_^-^’ and ‘NO_3_^-^’ condition when we released the constraints on the butyrate/acetate production ratio and the NH_4_^+^/NO_2_^-^ consumption ratio of *C. acetobutylicum* (Fig 6).

The model predicted that an optimal *C. acetobutylicum* would have a changed metabolism relative to the experimental data. For instance, acetate rather than butyrate should be produced when *C. acetobutylicum* grows optimally. This behaviour was seen both in the ‘NH_4_^+^ + NO_3_^-^’ and the ‘NO_3_^-^’ condition. The explanation is that more ATP can be generated from acetate production than from butyrate production. An optimal *C. acetobutylicum* would not consume NO_2_^-^ when growing at the ‘NH_4_^+^ + NO_3_^-^’ condition, because NO_2_^-^ reduction by nitrite reductase decreases growth of *C. acetobutylicum* according to the metabolic model. As explained, the amount of H_2_ produced is inversely related with the amount of NO_2_^-^ taken up. As a consequence, 159% more H_2_ was produced in the optimal scenario relative to the experiments in the ‘NH_4_^+^ + NO_3_^-^’ condition. A smaller increase in H_2_ production (+23%) was shown in the ‘NO_3_^-^’ condition, suggesting that the interactions between the two species were not optimal in the experiments at those two conditions. However, the experimental growth-rate of *C. acetobutylicum* was close to its maximal, computed value. For the ‘NH_4_^+^ + NO_3_^-^’ the experimental growth rate was 87% of the optimal value, whereas for the ‘NO_3_^-^’ this was 94%. These results suggest that the experimental behaviour of the community with imposed bidirectional interactions is closer to the optimal metabolic behaviour than a community with unidirectional interactions.

## Discussion

In this study we showed that the combination of a stoichiometric metabolic model with measured community-level concentrations and biomass abundances allows for the inference of metabolic interactions in a microbial community. This approach is complementary with current approaches that are based on correlation or co-occurrence networks, which predicts the community-interaction structure for large complex ecosystems [5, 41, 42]. These networks identify co-occurrence of species, suggesting that certain species in an ecosystem form a positive relationship with each other. However, the mechanisms behind the interactions remain unknown; here we infer them with metabolic models.

Stoichiometric models of metabolism make inference of metabolic exchange fluxes possible because they relate the uptake and production of environmental metabolites, to intracellular metabolic fluxes, organism abundance and growth rate. Such models are therefore integrating heterogeneous data types from which we make inferences about community behaviour. In addition to experimental data of species abundances and metabolite concentrations, molecular data can be used in the construction of stoichiometric metabolic models [43–45]. It is the integrative capability of stoichiometric models that makes them so useful for addressing current challenges in microbial ecology.

Metabolic models have been used to predict the behaviour of industrially relevant co-cultures and the type of interactions required to predict this behaviour [13, 14]. We show that this approach also works for ecologically relevant communities, where interspecies hydrogen transfer is the dominating interaction. Additionally, not only the type of interactions is identified, but we also infer the size of the interactions from the experimental data. With this method we show that the nitrogen source plays an important role in the rate of H_2_ exchange in our co-culture. Recently, Embree *et al.* [46] also inferred metabolic interactions of a microbial community, but optimized each species individually as a steady-state simulation and did not combine all metabolic models into one community. We, on the other hand, simulated the dynamics of the co-culture, because we fitted data from a dynamic system where species could change their metabolic behaviour during the experiment, which is closer to natural environments.

We have shown that our approach works for a co-culture and a next step would be the inference of metabolic interactions in a complex ecosystem. A complex community will have more interactions between the members of the community. In addition to beneficial relationships, competition can also play an important role in microbial communities [1]. The advantage of our dFBA based approach is that these type of interactions can also be inferred. Moreover, our results indicate that it is not even necessary to use full-blown genome-scale stoichiometric models for all members of a complex community as a coarse-grained model of *W. succinogenes* sufficed for the inference of metabolic interactions, as we recently suggested [7]. More detail can always be added later to refine predictions or resolve discrepancies, depending on the research question and data at hand.

Even though metabolic interactions can be inferred from experimental data, not all data and systems are equally amenable to a modeling approach. Firstly, a community with growing micro-organisms is advantageous. Growing cells have an active metabolism and increase their biomass in time, while interacting with other species. While non-growing species may also have an active metabolism, they do not increase in biomass, causing them to have lower fluxes, which makes it difficult to assign flux-values to such organisms. Secondly, controlled environments allow for more targeted perturbations of the community. Manipulation of the environment results in different dominating processes in the ecosystem [47]. In addition, a basic understanding of the core metabolism of the studied organisms is required. In our system, we had already an idea what type of interactions would take place, but this will not always be possible with complex ecosystems with poorly studied microorganisms. In such cases, metabolic reconstruction based on complete genomes is the best option to get a hint at the basic metabolic capacities. Lastly, a medium that is well-defined and minimal, is advantageous. Metabolites, which are turned over, are more easily identified in such media. A medium which, for instance, contains yeast extract provides all types of vitamins, amino acids and carbon compounds to the community members, and could mask or compromise the metabolic interactions in the ecosystem. With improved analytics, such challenges may be overcome, and we believe in view of the complexity of microbial communities, metabolic models are a promising -and arguable the only- tool for the inference of the metabolic interactions of co-cultures.

To conclude, in our eyes metabolic models are an indispensable tool for the inference of the metabolic interactions, as illustrated with our co-culture study. We have shown that the nitrogen-source quantitatively influences the interspecies hydrogen transfer, which was not feasible with experimental data alone. Moreover, the metabolic models can be used for more purposes than inference of metabolic interactions. They can also be used to infer the intracellular fluxes of species, which informs us about the metabolic adaptations of microorganisms, during different conditions. The models can also be used to investigate whether species in a community grow optimally or whether they are limited by external factors. This is particularly interesting for communities used for industrial processes, such as the anaerobic digestion process, where high biogas yields are desired. Finally, metabolic models could indicate strategies to steer the community in a desired direction, guiding community engineering approaches. Thus metabolic models have great potential –are simply required– to study complex, microbial ecosystems.

## Supporting information

S1 Material and Methods (PDF)

S1 Table A coarse-grained metabolic model of *W. succinogenes* was detailed enough to fit the experimental data. All metabolites, except for H^+^ and H_2_O, are element-balanced in the metabolic model.
(PDF)

S2 Table Parameters used for simulation of the co-culture. (PDF)

S1 Fig The dFBA simulations, with the draft genome-scale metabolic model of *W. succinogenes*, also agree mostly with the experimental data for the four different cultivation conditions. The metabolite profile plots contain error bars, but the other subplots not. The biomass ratios are based on the gene-copy data and the carbon balance consisted of the measured metabolites that contained carbon. The remaining missing carbon is assumed to be incorporated into biomass.
(EPS)

S2 Fig Product and substrate yields did not change during different growth rates. The extracted specific uptake and production rates (mmol/(gDW⋅h)) are plotted against the simulated growth rate *μ* (h-1). All lines are lineair what suggests that the specific uptake and production rates of both species are scaled with the growth. The N_2_ condition is not plotted, because the simulated growth rate was constant over the whole simulation.
(EPS)

S3 Fig Inference of the metabolic interactions using a draft genome-scale metabolic model of *W. succinogenes* showed only differences in the NO_2_^-^ production and NO_3_^-^ consumption per utilized H_2_. In **A** the calculated flux-values for *C. acetobutylicum* are normalized for the glucose uptake. **B** shows the calculated flux-values for *W. succinogenes* and are normalized for the H_2_ uptake. Note that H_2_ production by *C. acetobutylicum* is not equal to the specific H_2_ uptake rate of *W. succinogenes* as the biomass abundances should be taken into account, which were not equal during the experiment.
(EPS)

S1 File Draft genome-scale metabolic model of *W. succinogenes*. The reactions and the corresponding reversibility and bounds are listed in ‘reactions’. The metabolites-id and the corresponding names are listed in ‘metabolites’. All reactions and their corresponding substrates and products are listed in ‘network_react’, where in ‘network_metab’ all reactions per metabolite is listed. All metabolites and their corresponding MIRIAM information is listed in ‘miriam.’
(XLS)
