# Supplementary material for: Model-based quantification of metabolic interactions from dynamic microbial-community data
Source: PLoS One. 2017 Mar 9;12(3):e0173183. doi: 10.1371/journal.pone.0173183 (PMC5344373; doi:10.1371/journal.pone.0173183)
Supplement: S1 Material and Methods — (PDF) [file pone.0173183.s001.pdf]

Material & Methods

Simulating co-culture growth

Dynamic Flux Balance Analysis is based on Flux Balance Analysis (FBA) principles [1]. It is based on mass balances in steady state, expressed as S·v = 0 for each time interval: S represents the stoichiometric matrix of the reactions of the metabolic model, where the columns represent the reactions and the rows the metabolites, and v the flux-vector. Dynamics of the co-culture was simulated by using a Michaelis-Menten equation ($v_{upt} = V_{max}\frac{[S]}{K_m+[S]}$) for the substrate uptake reaction, where v_{upt} represents the uptake reaction, V_{max} the maximum rate of the uptake reaction, [S] the substrate concentration and K_m the substrate affinity. The parameters used in the Michaelis-Menten equation are listed in S2 Table. In each time interval a substrate uptake rate is calculated from the substrate concentration, and used as a flux constraint on the model; conversely, the substrate concentration is updated by the calculated fluxes. Under the assumption that the rate of change of extracellular substrates is much smaller than the intracellular fluxes, (pseudo-) steady states for intracelullar metabolism is applied at all times. The simulation consisted of 400 time points that resulted in a time interval of 3.3 minutes and created smooth metabolite profiles. Irreversible Michaelis-Menten kinetics were set for glucose-uptake by *C. acetobutylicum* and H_2-uptake by *W. succinogenes*. Biomass production was optimized during the simulations. V_{max} and K_m were adjusted to correctly simulate the experimental data. The initial concentrations in the simulations were equal to the initial measured concentrations. The ratio of butyrate and acetate production by *C. acetobutylicum* was constrained to agree with the experimental data and was done via manual fitting. The same was done for the ratio of NH_4^+ and NO_2^- consumption by *C. acetobutylicum*.

The following parameters were used for the simulation of co-culture growth during the different conditions. The scripts for each condition and the models are found online: `https://sourceforge.net/projects/cbmpy/files/publications/data/2017_Hanemaaijer/`.

Model construction of *W. succinogenes*

The following model for the metabolism of *W. succinogenes* was used (S1 Table). Biomass is differently defined in the experimental data (based on gene-copies) than in the model (based on gram dry weight (gDW)). From our modelling we concluded that one cell of *C. acetobutylicum* is 4.3 times bigger than cells of *W. succinogenes*, which is close to our experimentally observed 5.2 difference. Also the calculated C:N ratio of 1:0.23 for biomass of *W. succinogenes* is consistent for what is found in other bacteria [2, 3].

The draft genome-scale metabolic model was reconstructed with the in-house developed software called 'MetaDraft' which uses proteome information from GenBank and compared that to other curated genome-scale metabolic models (*Escherichia coli*, *Methanosarcina barkeri*, *Helicobacter pylori*, *Mycobacterium tuberculosis* and *Staphylococcus aureus*). Gaps in the draft version of the metabolic model were filled based on literature information. The metabolic model was capable to biosynthesize all building blocks to produce biomass from the components in the medium that we used in our experiments. The draft genome-scale metabolic model consists of 771 reactions and 1028 metabolites and the biomass reaction was a simplified biomass reaction of *Helicobacter pylori*. We chose this biomass, because *W. succinogenes* is closest related to *Helicobacter pylori* relative to the other three strains that were used for comparison.

Design of minimal medium

A suitable minimal medium was designed based on the Widdel's fresh water medium, supporting growth of the two organisms. This medium is suboptimal for *C. acetobutylicum*, because of the relatively high pH (7.0), and the omission of yeast extract or other growth-enhancing, but non-defined, components. This resulted in non-consistent growth rates of *C. acetobutylicum* in pure culture. In the presence of *W. succinogenes*, *C. acetobutylicum* growth however occurs readily and consistently. We therefore focused on the rates in the co-culture and not in pure culture. Probably, in a co-culture, *C. acetobutylicum* remains longer active than in co-culture, which results in rapid growth after propagation and is not the case in pure culture.

References

1. Mahadevan R, Schilling C. The effects of alternate optimal solutions in constraint-based genome-scale metabolic models. Metabolic engineering. 2003;5(4):264–276.

2. Goldman JC, Caron DA, Dennett MR. Regulation of gross growth efficiency and ammonium regeneration in bacteria by substrate C: N ratio1. Limnology and Oceanography. 1987;32(6):1239–1252.

3. Battley EH. Calculation of entropy change accompanying growth of Escherichia coli K-12 on succinic acid. Biotechnology and bioengineering. 1993;41(4):422–428.
