# Supplementary material for: Model-based quantification of metabolic interactions from dynamic microbial-community data
Source: PLoS One. 2017 Mar 9;12(3):e0173183. doi: 10.1371/journal.pone.0173183 (PMC5344373; doi:10.1371/journal.pone.0173183)
Supplement: S1 Table — All metabolites, except for H+ and H2O, are element-balanced in the metabolic model. (PDF) [file pone.0173183.s002.pdf]

**Reaction name**	**Reaction**
H_2 transport	$H_2 \rightarrow H_2(e)$
NO_2^- transport	$NO_2^- \rightarrow NO_2^-(e)$
NO_3^- transport	$NO_3^- \rightarrow NO_3^-(e)$
Acetate transport	$\text{Acetate} + H^+ \rightarrow \text{Acetate(e)} + H^+(e)$
NO_3^- reductase	$2NO_3^- + 2QH2 \rightarrow 2NO_2^- + ATP + 2Q + 2H_2O$
NO_2^- reductase	$NO_2^- + 3QH2 + 2H^+ \rightarrow NH_4^+ + 3Q + 2H_2O$
H_2 reductase	$H_2 + Q \rightarrow QH2$
Biomass Reaction	$30ATP + 2.2NH_4^+ + 4.84\text{Acetate} \rightarrow \text{Biomass}$
