# Supplementary material for: Model-based quantification of metabolic interactions from dynamic microbial-community data
Source: PLoS One. 2017 Mar 9;12(3):e0173183. doi: 10.1371/journal.pone.0173183 (PMC5344373; doi:10.1371/journal.pone.0173183)
Supplement: S2 Table — (PDF) [file pone.0173183.s003.pdf]

**Parameter**	$\mathbf{NH_4^+ + NO_3^-}$	$\mathbf{NH_4^+}$	$\mathbf{NO_3^-}$	$\mathbf{N_2}$
V_{max} Glc uptake *C. acetobutylicum*	16	16	15	19
K_m Glc uptake *C. acetobutylicum*	2	2	2	2
V_{max} H_2 uptake *W. succinogenes*	55	55	55	55
K_m H_2 uptake *W. succinogenes*	0.001	0.001	0.001	0.001
Initial biomass *C. acetobutylicum* (g)	0.0009	0.0009	0.0009	0.0009
Initial biomass *W. succinogenes* (g)	0.0003	0.0003	0.0003	0.0003
Butyrate/acetate flux ratio	0.17	2.23	0.12	2.16
NH_4^+/NO_2^- ratio	0.20	-	-	-
